# *Salicornia europaea* L. as a Marine Bioactive Resource: Phytochemical Profile, Health Mechanisms, and Functional Applications in Precision Nutrition

**DOI:** 10.3390/md24070229

**Published:** 2026-06-29

**Authors:** José Francisco Tornero-Aguilera, Carlota Valeria Villanueva-Tobaldo, Edgar Simón Sancho-Haro, Mario Muñoz-López, Miguel López-Moreno, Rodrigo Yáñez-Sepúlveda, José Francisco López-Gil, Vicente Javier Clemente-Suárez

**Affiliations:** 1 Department of Sport Sciences, Faculty of Sport and Health Sciences, Fit Generation Research Institute, AD500 Andorra la Vella, Andorra; jtornero@fitgeneration.es (J.F.T.-A.); mario.mlopez@fitgeneration.es (M.M.-L.); 2 Department of Nutrition and Dietetics, Faculty of Sport and Health Sciences, Fit Generation Research Institute, AD500 Andorra la Vella, Andorra; c.vtobaldo@gmail.com (C.V.V.-T.); edgar.sancho@fitgeneration.es (E.S.S.-H.); 3 Institute of Health and Sport Sciences, Faculty of Health Sciences, Universidad Francisco de Vitoria, 28223 Madrid, Spain; nutreconciencia.blog@gmail.com; 4 Facultad de Educación y Ciencias Sociales, Universidad Andrés Bello, Viña del Mar 2200055, Chile; rodrigo.yanez.s@unab.cl; 5 School of Medicine, Universidad Espíritu Santo, Samborondón 092301, Ecuador; josefranciscolopezgil@gmail.com; 6 Faculty of Health Sciences, Universidad Autónoma de Chile, Temuco 4780000, Chile

**Keywords:** *Salicornia europaea*, phytochemicals, polyphenols, isorhamnetin, functional food, cardiovascular health, metabolic syndrome, precision nutrition, gut microbiome, prebiotic

## Abstract

Marine halophytes are gaining attention as a source of plant-derived bioactive compounds with potential applications across nutraceuticals, functional foods, and preventive nutrition. Among them, *Salicornia europaea* L. is a coastal succulent whose adaptation to hypersaline environments shapes a distinctive phytochemical profile of pharmacological interest. This narrative review integrates current evidence on the bioactive composition, mechanistic activities, and translational relevance of *S. europaea* and related *Salicornia* species. Their secondary metabolome includes flavonols, isorhamnetin glycosides, hydroxycinnamic acids, oleanane-type triterpene saponins, fermentable polysaccharides, carotenoids, and a mineral-rich ionic matrix. Reported activities span antioxidant, anti-inflammatory, vascular-protective, anti-adipogenic, glycaemic-modulating, antimicrobial, and microbiome-related effects, mediated through pathways involving NF-κB, PPAR-γ, endothelial nitric oxide signalling, and short-chain fatty acid production. Beyond its individual phytochemical components, the matrix as a whole may also support sodium-reduction strategies in food formulation, providing a complementary nutritional rationale for its incorporation as a functional ingredient. Despite a coherent body of mechanistic and preclinical findings, clinical evidence remains limited, particularly regarding long-term efficacy, dose standardisation, and bioavailability in humans. Future work should prioritise adequately powered intervention trials and standardised characterisation of marine halophyte bioactives to clarify their evidence-based role in functional food development and future precision nutrition applications.

## 1. Introduction

The pandemic of non-communicable diseases (NCDs)—cardiovascular disease (CVD), type 2 diabetes mellitus (T2DM), obesity, hypertension, and metabolic syndrome—represents the dominant cause of global morbidity and mortality. The Global Burden of Disease (GBD) 2021 Risk Factors Collaborators analysis confirmed that suboptimal diets remain a leading driver of global disease burden, with high sodium intake among the top dietary risk factors and contributing to several million deaths annually [1]. The World Health Organization recommends daily sodium consumption below 2000 mg, yet population surveillance data indicate that average intake across most world regions exceeds 3500 to 4500 mg per day, representing an excess of 75–125% above recommended thresholds [2,3]. Dietary sodium reduction has accordingly been identified as one of the most cost-effective public health interventions available, with modelled analyses suggesting that a 30% reduction in sodium intake across populations would prevent approximately 900,000 deaths from CVD annually in low- and middle-income countries alone [3].

The mechanistic link between sodium excess and cardiovascular pathology is well characterised. The Intersalt Cooperative Research Group study, including 10,079 participants across 32 countries, demonstrated a dose–response relationship between 24-h urinary sodium excretion and systolic blood pressure across all age groups, providing population-level evidence for the sodium–hypertension axis [4]. Subsequent meta-analyses, including the systematic review by Aburto et al. covering 36 randomised controlled trials and 12 cohort studies, confirmed that a reduction in sodium intake of approximately 2000 mg/day was associated with a mean reduction in systolic blood pressure of 3.4 mmHg and in diastolic blood pressure of 1.5 mmHg [5]. The clinical significance of this magnitude of reduction is substantial at the population level, with a 2 mmHg reduction in systolic blood pressure estimated to reduce stroke mortality by approximately 10% and coronary heart disease mortality by 7% in middle-aged adults [6]. The Salt Substitute and Stroke Study (SSaSS), a 2021 cluster-randomised trial encompassing 20,995 participants across 600 villages in rural China, demonstrated that replacing 25% of sodium chloride with potassium chloride reduced the risk of stroke by 14%, total CVD events by 13%, and all-cause mortality by 12%, providing the most compelling clinical evidence to date for salt substitution strategies [7].

Conventional sodium reduction strategies—including mandatory reformulation, consumer education, and KCl-based substitution—encounter substantial practical limitations. Potassium chloride, the most widely deployed sodium substitute, introduces a bitter metallic off-note at concentrations above 20–30% replacement, limiting its palatability and consumer acceptance [8]. Synthetic taste modifiers and flavour maskers, while technically effective, conflict with the clean-label positioning increasingly demanded in health-oriented food markets. These limitations have motivated a growing scientific interest in naturally sourced, bioactive-rich alternatives that may contribute to sodium reduction while delivering complementary nutritional benefits compatible with current regulatory frameworks [8,9]. Marine ecosystems represent an underexplored source of such ingredients, with coastal halophytes positioned at the intersection of sustainable agronomy, food technology, and preventive nutrition.

Beyond the specific question of sodium reduction, the rationale for marine halophytes as functional ingredients ultimately rests on their broader phytochemical complexity. Their adaptation to hypersaline environments drives the accumulation of secondary metabolites—flavonoids, hydroxycinnamic acids, isorhamnetin glycosides, triterpene saponins, and fermentable polysaccharides—whose biological activities extend across antioxidant, anti-inflammatory, vascular, metabolic, and microbiome-related axes. It is this multi-target bioactive profile, rather than sodium substitution alone, that defines the translational interest of *Salicornia europaea* within the present review.

Marine organisms and marine-derived natural products have become one of the most productive frontiers for the discovery of effective biological agents, yielding metabolites with well-documented antioxidant, anti-inflammatory, and anticancer activities that are now actively pursued for pharmaceutical and nutraceutical development [10,11,12]. Recent syntheses have underscored the therapeutic potential of marine-derived antioxidants in oxidative-stress-associated diseases [11], the anti-inflammatory capacity of marine metabolites [10], and the rapidly expanding pipeline of marine bioactives advancing toward cancer therapy [12]. Within this marine pharmacopoeia, coastal halophytes occupy a distinctive niche: unlike most marine bioactive sources, which are algal, microbial, or invertebrate in origin, *Salicornia europaea* is a vascular plant that can be cultivated agronomically while retaining a marine-shaped phytochemistry, positioning it as a tractable and scalable source of marine bioactives for human health.

*Salicornia europaea* L. (family Amaranthaceae, formerly Chenopodiaceae), commonly known as glasswort, marsh samphire, or sea asparagus, is a succulent annual halophyte distributed across coastal salt marshes, intertidal mudflats, sea cliffs, and inland saline soils throughout the Atlantic and Mediterranean coasts of Europe, North Africa, and Central Asia. Its evolutionary adaptation to substrate salinities of 10 to 100 g NaCl per litre has endowed the plant with a distinctive phytochemical profile associated with tolerance to osmotic and oxidative stress, as well as the accumulation of bioactive compounds with potential relevance to inflammation, glycation, and chronic disease-related pathways in humans [13,14]. The secondary metabolome of *S. europaea* includes polyphenols, including flavonoids, with potent antioxidant and anti-inflammatory activities, isorhamnetin glycosides associated with anti-adipogenic and lipid-regulatory properties, phenolic acids with vascular-protective effects, oleanane triterpene saponins exhibiting lipase-inhibitory activity, and fermentable dietary fibre supporting gut microbiome function [15,16,17,18]. Its mineral matrix simultaneously delivers a naturally balanced electrolyte profile with substantially reduced bioavailable sodium relative to crystalline NaCl, supporting its potential use as a functional condiment ingredient rather than a simple sodium analogue.

Despite its distinctive bioactive profile, *Salicornia* remains substantially underrepresented in the broader literature on marine-derived functional ingredients relative to its pharmacological and nutritional potential. The existing literature is fragmented across ethnobotany, agronomy, food chemistry, and pharmacology journals, with limited integration of phytochemical profile, mechanistic health evidence, and translational applications within a marine biotechnology framework. This narrative review aims to address this gap by: (i) summarising the published research that has characterised the marine bioactive phytochemical portfolio of *S. europaea* and related *Salicornia* species; (ii) reviewing and integrating preclinical and available clinical evidence for health-promoting properties across cardiovascular, metabolic, antioxidant, anti-inflammatory, and gut microbiome axes; (iii) examining translational applications in functional food development; (iv) summarising the international regulatory landscape; and (v) identifying research priorities limiting clinical translation. The review is positioned within the scope of marine-derived bioactives for human health, at the interface of food science, functional ingredient development, precision nutrition, and preventive medicine. To our knowledge, this is the first review to integrate, within a single marine-biotechnology framework, the phytochemical profile, mechanistic health evidence, regulatory landscape, and documented functional-food and clinical applications of *Salicornia europaea*, explicitly linking the plant’s halophytic stress physiology to its translational value as a multi-target marine bioactive resource for precision nutrition; this integrative, mechanism-to-application perspective constitutes the principal novelty of the present work.

## 2. Materials and Methods: Literature Search Strategy

This narrative review was conducted following the methodological principles for narrative reviews of biomedical literature recommended by the Cochrane Collaboration and established precedents in nutrient and food science review literature. Although a narrative review design does not require a formal Preferred Reporting Items for Systematic Reviews and Meta-Analyses (PRISMA) reporting, a structured search strategy was included to ensure systematic literature identification. The search was conducted between January 2024 and January 2026, with reference checking updated to April 2026 to capture recently published studies.

Systematic electronic searches were performed across four databases: PubMed/MEDLINE, Scopus, Web of Science Core Collection, and Google Scholar. The primary search strategy combined the terms (*Salicornia*) OR (glasswort) OR (sea asparagus) OR (marsh samphire) OR (halophyte food) with secondary keywords related to the domains of phytochemistry, biological activity, functional food applications, and disease-relevant mechanisms. Supplementary Medical Subject Headings (MeSH) terms employed included: *Salicornia*; Plants, Medicinal; Flavonoids/pharmacology; Phenols/analysis; Antioxidants/therapeutic use; Sodium, Dietary/adverse effects; Cardiovascular Diseases/prevention and control; Gastrointestinal Microbiome; Adipogenesis; PPAR gamma; Fermentation. Additional literature was identified through manual screening of reference lists from retrieved articles. Grey literature sources, including reports from regulatory agencies such as the European Food Safety Authority (EFSA), the U.S. Food and Drug Administration (FDA), the Korean Ministry of Food and Drug Safety (MFDS), and the World Health Organization, were also considered when relevant to regulatory or translational aspects.

Inclusion criteria were: (a) peer-reviewed original research articles, systematic reviews, meta-analyses, or narrative reviews; (b) studies reporting on the phytochemical composition, biological activities, health effects, food technology applications, or regulatory status of *Salicornia* spp. or closely related halophyte species; (c) studies employing in vitro, in vivo animal, or human clinical designs; (d) mechanistic studies involving key bioactive constituents relevant to *Salicornia* phytochemistry, including flavonols, isorhamnetin, ferulic acid, and saponins; and (e) publications in English or Spanish. Exclusion criteria included unpublished data, conference abstracts without full-text availability, studies using *Salicornia* exclusively as a model organism for salinity tolerance without bioactive or nutritional relevance. Priority was assigned to human clinical evidence, followed by well-controlled in vivo animal studies, while in vitro findings were used primarily to support mechanistic interpretation.

## 3. Botanical Profile, Phylogenetics, and Halophytic Ecology of *Salicornia europaea*

*Salicornia* (Tourn.) L. belongs to the tribe Salicornieae within the family Amaranthaceae (subfamily Salicornioideae) and comprises approximately 30–40 accepted species, though the taxonomy within the genus remains challenging due to high morphological plasticity, extensive hybridisation, and polyploidy. The comprehensive phylogenetic analysis by Kadereit et al. resolved major infrageneric relationships, separating annual (*Salicornia* s.s.) from perennial (*Sarcocornia*) lineages, and clarifying the phylogenetic positions of the most biologically relevant taxa [13]. The most extensively studied taxa include *S. europaea*, *S. herbacea*, *S. bigelovii*, and *S. ramosissima*, which collectively represent the principal pharmacological and nutritional models investigated within the genus. Despite species-level differences in flavonoid profiles, the bioactive compound architecture—mineral accumulation strategy, flavonol/phenolic acid complement, isorhamnetin glycosides, and dietary fibre—is sufficiently conserved across the genus to justify an integrated review approach. Nevertheless, findings derived from related *Salicornia* or *Sarcocornia* species should be interpreted as supportive rather than fully substitutive evidence for *S. europaea*, with species-level variations noted where documented [13,18].

The ecological adaptation of *Salicornia* spp. to hypersaline environments is closely linked to the accumulation of secondary metabolites involved in osmotic regulation, oxidative stress defence, and ion homeostasis. These adaptive pathways drive the biosynthesis of flavonoids, phenolic acids, osmoprotectants, and antioxidant compounds that may also exert biological activity in human systems.

*S. europaea* is an obligate annual, germinating from late winter to early spring, completing its life cycle in 60 to 90 days, and senescing with the arrival of autumn frosts. It occupies intertidal salt marsh communities, sea cliffs, and inland saline and alkaline soils throughout its native range, growing in substrate NaCl concentrations that are lethal to the vast majority of glycophyte crops. The plant grows optimally at 100–200 mM NaCl but tolerates concentrations of up to 1000 mM—a halophytic capacity ranking among the highest of any angiosperm [14]. This extraordinary salinity tolerance operates through a suite of coordinated physiological and biochemical mechanisms: vacuolar compartmentalisation of Na^+^ and Cl^−^ through the action of tonoplast Na^+^/H^+^ antiporters (NHX family); synthesis of compatible osmolytes including glycine betaine, proline, and mannitol to maintain osmotic equilibrium in the cytoplasm; upregulation of antioxidant enzyme systems (superoxide dismutase, catalase, ascorbate peroxidase, glutathione reductase) to neutralise the elevated reactive oxygen species (ROS) generated by ionic stress; and production of secondary metabolite ultraviolet (UV) and ROS shields—the polyphenolic compounds whose pharmacological properties are the central subject of this review [14,19].

The direct biochemical link between the stress-adaptive secondary metabolome of *S. europaea* and its pharmacological relevance to human health is conceptually relevant for halophyte functional food research. Many of the flavonoids and hydroxycinnamic acids produced in response to UV radiation and oxidative stress are structurally analogous to dietary polyphenols associated with antioxidant, anti-inflammatory, and cardioprotective effects in human epidemiological and clinical studies. The mineral matrix—with elevated potassium, magnesium, and calcium and partially compartmentalised sodium—reflects the plant’s adaptation to ionic homeostasis under salinity stress and aligns with mineral profiles considered favourable in sodium-excess dietary patterns [9,16]. This overlap between plant ecophysiology and human nutritional pharmacology underpins the interest in *Salicornia* as a candidate functional ingredient, where the bioactive profile arises directly from coastal evolutionary adaptation rather than from compositional fortification.

Mediterranean and Atlantic commercial cultivation of *S. europaea* has expanded substantially in the past decade, with established growing operations in Catalonia and Valencia (Spain), the Wadden Sea region (Netherlands), Brittany and Camargue (France), and emerging pilot operations in Portugal and Morocco. Agronomic advantages are considerable: the plant tolerates marginal coastal and inland saline soils otherwise unsuitable for conventional agriculture, can be irrigated with brackish water or diluted seawater conserving freshwater resources, requires minimal agrochemical inputs owing to its tolerance of hypersaline conditions that inhibit most pathogens and weeds, and yields two to three harvests per growing season [8,20]. Life cycle assessments of Mediterranean *Salicornia* salt production have consistently indicated substantially lower carbon, water, and land footprints compared with conventional mineral salt mining, KCl extraction, or terrestrial vegetable cultivation on irrigated land, supporting its sustainability profile within functional ingredient applications [8]. In the Gulf Cooperation Council region, pilot projects coordinated by the International Center for Biosaline Agriculture (ICBA, Dubai, UAE) have validated *Salicornia* cultivation as a viable desert agroecology strategy using saline groundwater or treated wastewater for irrigation, simultaneously addressing food security, functional ingredient supply, and saline land rehabilitation goals [8,9].

## 4. Bioactive Phytochemical Profile of *Salicornia europaea*

The secondary metabolome of *Salicornia europaea* encompasses diverse bioactive classes including flavonoids, phenolic acids, isorhamnetin glycosides, triterpene saponins, carotenoids, and a mineral-rich ionic matrix, with bioactivity profiles extending from antioxidant and anti-inflammatory to anti-adipogenic, antimicrobial, and prebiotic [18]. Chromatographic analyses employing high-performance liquid chromatography with diode-array detection (HPLC-DAD), liquid chromatography–tandem mass spectrometry (LC-MS/MS), and nuclear magnetic resonance (NMR) spectroscopy have identified flavonols, flavanones, phenolic acids, isorhamnetin glycosides, chromones, triterpenoid saponins, carotenoids, and a mineral-rich ionic matrix as the major bioactive classes. Importantly, the concentrations and profiles of these constituents vary as a function of growing substrate salinity, harvest timing, plant part (aerial vs. root), geographic ecotype, and post-harvest processing conditions—variability that has significant implications for standardisation in functional food applications [9,18]. Table 1 provides an integrated overview of the major bioactive classes, representative concentration ranges, and documented bioactivities. More specifically, the flavonol and isorhamnetin glycoside fractions have been resolved chiefly by HPLC-DAD and LC-MS/MS, the hydroxycinnamic acids by LC-MS/MS, the triterpene saponins by LC-MS/MS and NMR, and the ionic matrix by ICP-OES/ICP-MS; the representative concentration ranges for each compound class, together with the corresponding analytical sources, are compiled compound-class by compound-class in Table 1 [9,17,18].

### 4.1. Flavonoids and Other Polyphenols

A terminological clarification is warranted: flavonoids are themselves a subclass of polyphenols, and the two terms are therefore not mutually exclusive. Throughout this review the term polyphenol is reserved for compounds bearing more than one phenolic ring or hydroxyl-substituted ring (i.e., flavonoids and related multi-ring structures), whereas the simpler hydroxycinnamic and hydroxybenzoic acids, which carry a single phenolic ring, are designated phenolic acids rather than polyphenols sensu stricto.

Flavonols constitute the dominant and pharmacologically most relevant class of secondary metabolites in *S. europaea*. Quercetin (3,3′,4′,5,7-pentahydroxyflavone) and its glycoside conjugates—quercetin-3-*O*-glucoside (isoquercitrin), quercetin-3-*O*-rutinoside (rutin), and quercetin-3-*O*-galactoside—are typically present at 1 to 5 mg/g dw in aerial parts, with peak concentrations reported at the vegetative growth stage prior to anthesis [9,18]. Quercetin is among the most extensively studied dietary flavonoids in biomedical research. In a prospective cohort study conducted in Zutphen, Netherlands, Hertog et al. observed that flavonol intake was inversely associated with mortality from coronary heart disease in 805 middle-aged men followed for five years (relative risk 0.32 for highest vs. lowest quintile), a finding that motivated subsequent mechanistic and clinical investigation [28]. The mechanisms underlying quercetin’s cardiovascular activity include inhibition of low-density lipoprotein (LDL) oxidation through both direct radical scavenging and upregulation of paraoxonase-1 (PON-1), a high-density lipoprotein (HDL)-associated enzyme that hydrolyses lipid peroxides; inhibition of platelet aggregation through modulation of arachidonic acid metabolism; and suppression of endothelial inflammation via NF-κB pathway attenuation [29,30].

Kaempferol(3,4′,5,7-tetrahydroxyflavone)and its glycosides, including kaempferol-3-*O*-glucoside and kaempferol-3-*O*-rutinoside, contribute additional antioxidant and estrogenic modulation effects in *Salicornia* extracts. Kaempferol has demonstrated anti-inflammatory activity in multiple experimental systems through dual inhibition of cyclooxygenase-2 (COX-2) and lipoxygenase pathways, and has been shown to suppress VEGF-mediated angiogenesis in endothelial cell models—a property potentially relevant to preventing the neovascularisation associated with proliferative diabetic retinopathy and tumour growth [29,31]. Luteolin (3′,4′,5,7-tetrahydroxyflavone), present in lesser quantities in *S. europaea* aerial parts, provides complementary anti-inflammatory activity through inhibition of pro-inflammatory cytokine production (TNF-α, IL-6, IL-1β) and inhibition of nitric oxide synthase in activated macrophages. Myricetin, present in trace quantities, contributes to manganese superoxide dismutase (MnSOD) upregulation and mitochondrial protection [31].

The total polyphenol content (TPC) of *S. europaea* aerial parts, measured by Folin–Ciocalteu assay, typically ranges 8 to 15 mg gallic acid equivalents (GAE)/g dw, with systematic inter-species variation across the genus: *S. herbacea* (East Asian ecotype) consistently reports the highest values at 10 to 12 mg GAE/g dw, while *S. bigelovii* exhibits more variable and generally lower values at 5 to 8 mg GAE/g dw [17,18]. The total flavonoid content (TFC) determined by aluminium chloride colorimetry ranges 4 to 10 mg quercetin equivalents (QE)/g dw, with high correlation between TPC and TFC (r typically 0.85 to 0.95) confirming flavonols as the dominant polyphenolic fraction [9,18]. These values compare favourably with common culinary herbs such as oregano, sage, and rosemary, and with other marine-origin botanical ingredients including brown algae extracts and seagrass-derived phenolics: DPPH IC_50_ values of 0.3 to 0.9 mg/mL reported for *Salicornia* extracts are comparable to those of rosemary, green tea, and grape marc, and substantially exceed those described for several conventional plant-derived antioxidant sources [9,17].

### 4.2. Isorhamnetin Glycosides: Anti-Adipogenic Marine Bioactives

Isorhamnetin(3′-methoxy-3,4′,5,7-tetrahydroxyflavone; 3′-methylquercetin)and its principal glycoside conjugates, isorhamnetin-3-*O*-glucoside and isorhamnetin-3-*O*-rutinoside, are among the most pharmacologically distinctive constituents of *Salicornia* spp. Reported at concentrations of 0.5 to 2 mg/g dw in aerial parts of *S. herbacea* and at lower but significant concentrations in *S. europaea*, these compounds represent a relevant point of phytochemical differentiation between halophyte-derived polyphenols and those of conventional terrestrial flavonoid sources [15,18]. The mechanistic characterisation of isorhamnetin-3-*O*-glucoside as an anti-adipogenic agent was established by Park et al., who demonstrated dose-dependent inhibition of adipocyte differentiation in the 3T3-L1 preadipocyte model with a 40 to 60% reduction in lipid droplet accumulation at physiologically accessible concentrations of 10 to 50 μM [15]. The molecular mechanism operates through suppression of peroxisome proliferator-activated receptor gamma (PPAR-γ), C/EBPα, and their downstream adipogenic transcription network, including fatty acid binding protein 4 (FABP4), perilipin-1, and sterol regulatory element-binding protein 1c (SREBP-1c) [15,32].

PPAR-γ is the master transcription regulator of adipogenesis and a critical integrator of lipid metabolism, glucose homeostasis, and inflammatory signalling in adipose tissue. Its activation by lipophilic ligands drives preadipocyte-to-adipocyte differentiation and promotes lipid storage, while pharmacological antagonism—the mechanism exploited by the thiazolidinedione class of anti-diabetic drugs—improves insulin sensitivity and reduces visceral adiposity [32]. The anti-PPAR-γ activity of isorhamnetin glycosides from *Salicornia* represents a structurally distinct, natural-origin modulation of this axis, relevant not only to obesity prevention but to the management of insulin resistance, non-alcoholic fatty liver disease (NAFLD), and the visceral adiposity component of metabolic syndrome [33,34]. Subsequent work by Lv et al. extended this mechanistic picture, demonstrating that isorhamnetin supplementation in high-fat diet mouse models attenuated hepatic steatosis through dual inhibition of de novo lipogenesis (suppression of FASN and SREBP-1c) and reduction in hepatic oxidative stress, with concomitant improvement in alanine aminotransferase (ALT) and aspartate aminotransferase (AST) levels, supporting translation to NAFLD contexts [21]. The bioavailability of isorhamnetin glycosides from food matrices involves intestinal hydrolysis by brush border glycosidases and colonic microbial *β*-glucosidases to yield the aglycone, followed by absorption and hepatic methylation of quercetin to generate isorhamnetin as a metabolite, creating a dual dietary and endogenous source of this bioactive form [35,36].

### 4.3. Phenolic Acids: Vascular-Protective Hydroxycinnamic Derivatives

Phenolic acids—specifically the hydroxycinnamic acid subclass—represent the second major phenolic (non-flavonoid) fraction of *S. europaea* and carry perhaps the most directly relevant vascular-protective mechanism among the plant’s bioactives. Ferulic acid (4-hydroxy-3-methoxycinnamic acid), *p*-coumaric acid, caffeic acid, sinapic acid, chlorogenic acid (5-caffeoylquinic acid), and protocatechuic acid have all been identified by LC-MS/MS analysis of *Salicornia* extracts, with ferulic acid and caffeic acid predominating [18,22,23,37]. Total phenolic acid content typically represents 30 to 50% of the total TPC determined by Folin–Ciocalteu assay in aqueous extracts, with higher proportions in methanolic and ethyl acetate fractions [17,18]. It should be noted that these hydroxycinnamic and hydroxybenzoic acids possess a single phenolic ring and are, strictly speaking, phenolic acids rather than polyphenols; the term polyphenol is therefore not applied to this fraction.

Ferulic acid has attracted particular attention in the context of *Salicornia*’s cardiovascular activity. A mechanistically rigorous study by Kim et al. demonstrated that *S. europaea* aqueous extract at concentrations providing an equivalent sodium load to NaCl did not reproduce the vascular dysfunction—specifically, impaired endothelium-dependent relaxation in isolated aortic ring preparations—observed under equivalent NaCl exposure [16]. Trans-ferulic acid was identified as the principal mediator of this vascular protection, acting through enhanced endothelial nitric oxide synthase (eNOS) phosphorylation at Ser1177, increased endothelial NO bioavailability, and reduced superoxide radical production via NADPH oxidase inhibition [16,22]. These observations are relevant to the design of functional sodium substitution strategies, suggesting that *Salicornia*-derived sodium delivers a functionally distinct vascular profile compared with crystalline NaCl, in which matrix-bound minerals and accompanying polyphenols may contribute vascular effects beyond the reduction in sodium dose. Ferulic acid’s gut microbial metabolite dihydroferulic acid has additionally been shown to inhibit angiotensin-converting enzyme (ACE) in vitro, suggesting a potential contribution to blood pressure regulation that parallels the mechanism of ACE-inhibitor antihypertensive drugs [22,23]. Caffeic acid exerts complementary anti-inflammatory and antioxidant effects through Nrf2/HO-1 pathway activation, while chlorogenic acid has been associated with attenuation of postprandial glycaemia through inhibition of intestinal glucose transport (SGLT1 and GLUT2), in line with the *α*-glucosidase inhibitory activity of the flavonol fraction [22,23].

### 4.4. Oleanane Triterpene Saponins

Oleanane-type triterpenoid saponins, including glycosides of oleanolic acid and hederagenin, represent a quantitatively minor but biologically significant fraction of *Salicornia*’s secondary metabolome, with concentrations that vary considerably across species and growing conditions [24,25]. These amphiphilic compounds exhibit several well-characterised pharmacological activities relevant to metabolic and cardiovascular health. Pancreatic lipase inhibition—the mechanism exploited by the anti-obesity drug orlistat—has been demonstrated for saponin-enriched fractions of *Salicornia* with IC_50_ values of approximately 0.3 mg/mL in enzymatic assays, suggesting capacity to reduce dietary fat absorption and attenuate postprandial lipid excursions at physiologically achievable concentrations [25]. Bile acid binding capacity, established for oleanane saponins more broadly through colonic-model studies, provides an additional lipid-lowering mechanism by reducing cholesterol micellar solubility and promoting faecal sterol excretion—a mechanism analogous to that of soluble dietary fibre and plant sterols [24]. Anti-inflammatory properties of triterpenoid saponins—including inhibition of COX-2 and 5-lipoxygenase, suppression of NF-κB, and attenuation of NLRP3 inflammasome activation—have been demonstrated in macrophage and endothelial cell models, providing mechanistic synergy with the flavonoid anti-inflammatory effects described above [24,30].

### 4.5. The Marine Mineral Matrix

**Figure 1:**
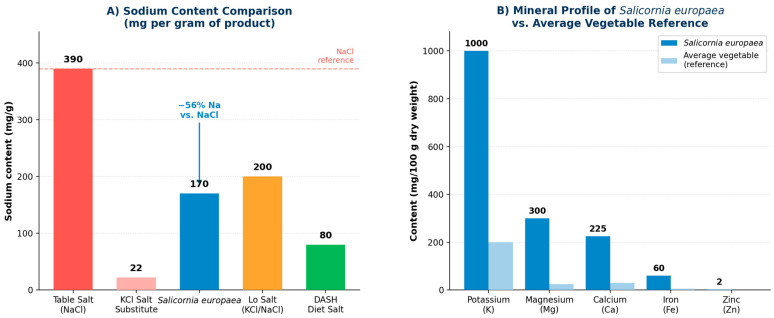
(**A**) Sodium content comparison across salt and condiment alternatives expressed per 100 g dry weight. (**B**) Mineral profile of *Salicornia europaea* compared with average vegetable reference values. K = potassium; Mg = magnesium; Ca = calcium; Fe = iron; Zn = zinc. Data represent representative ranges from peer-reviewed literature. dw = dry weight.

The mineral composition of *S. europaea* contributes to its nutritional and functional profile: a naturally balanced electrolyte matrix with substantially reduced bioavailable sodium relative to conventional table salt, while delivering complementary minerals whose deficiencies are prevalent in high-sodium, low-potassium Western dietary patterns. Published analytical data from multiple independent studies consistently report: potassium 800 to 1200 mg/100 g dw (approximately 5-fold higher than most common vegetables and comparable to bananas on a dry weight basis); magnesium 200 to 400 mg/100 g dw (comparable to pumpkin seeds and dark chocolate); calcium 150 to 300 mg/100 g dw; non-haem iron 40 to 80 mg/100 g dw (substantial but with absorption limited by the non-haem form and the presence of oxalates); and zinc 2 to 5 mg/100 g dw (Figure 1) [9,16].

Total sodium content of whole-plant *S. europaea* ranges from 2000 to 6000 mg/100 g dw depending on substrate salinity, harvest timing, and washing protocol. This wide range has occasionally been misinterpreted as contradicting the sodium reduction claim for *Salicornia*-derived salt. A mechanistically relevant distinction may be not only total sodium content but also matrix-dependent bioavailability and the co-delivery of vascular-relevant bioactives. Sodium in *S. europaea* is sequestered within plant cell vacuoles in ionic and partially organo-chelated forms, rather than as free crystalline NaCl. Preclinical studies examining urinary sodium excretion after *Salicornia* extract administration at matched sodium load versus NaCl have reported lower systemic sodium availability for the plant-matrix form, with reductions of approximately 40 to 50% in the available animal models [16], attributable to incomplete vacuolar release during gastrointestinal digestion, competitive absorption with the abundant potassium (which promotes natriuresis via intestinal exchanger competition), and the blunted intestinal sodium transport efficiency in the presence of polyphenols [5,16]. The potassium-to-sodium ratio of *S. europaea* (K:Na typically >0.25 to 0.5 across ecotypes and processing conditions) contrasts sharply with that of crystalline NaCl (K:Na = 0) and of KCl substitutes (which introduce off-flavour without the full mineral matrix), and compares favourably with the K:Na ratio associated with cardiovascular protection in epidemiological studies [38,39,40].

### 4.6. Dietary Fibre and Prebiotic Polysaccharides

*Salicornia* aerial parts contain 15 to 25 g/100 g dw of total dietary fibre, comprising both insoluble cellulosic and hemicellulosic fractions (70–75% of total fibre) and a fermentable soluble fraction (25–30%) including pectin-like galacturonans, soluble *β*-mannans, xyloglucans, and arabinoxylan-type polysaccharides [26,27]. The soluble fibre fraction is of particular functional interest: ex vivo faecal fermentation studies have demonstrated selective stimulation of *Bifidobacterium* spp. and *Lactobacillus* spp. populations, with prebiotic index values comparable to commercial inulin-type fructans at equivalent doses. Short-chain fatty acid (SCFA) production in fermentation models—primarily acetate, propionate, and butyrate—is substantial, with butyrate representing approximately 20 to 30% of total SCFA generated [26,27,41]. These polysaccharides also contribute viscosity-forming capacity in aqueous food systems, which provides technological utility for texture modification and, in the context of glycaemic management, delays gastric emptying and reduces postprandial glucose absorption velocity [27].

## 5. Antioxidant Capacity and Phenolic Characterisation

**Figure 2:**
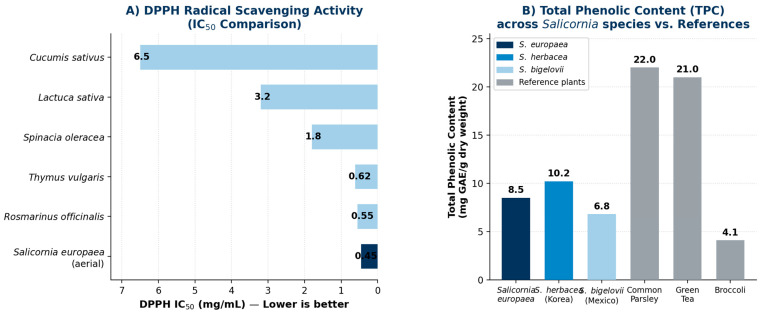
Antioxidant capacity of *Salicornia* spp. compared with common food plants. (**A**) DPPH radical scavenging IC_50_ (mg/mL; lower values indicate stronger activity). (**B**) Total phenolic content (TPC, mg GAE/g dry weight) across *Salicornia* species and reference plants. Data represent ranges from peer-reviewed literature. GAE = gallic acid equivalents; dw = dry weight.

Comprehensive antioxidant activity of *S. europaea* and related *Salicornia* species has been evaluated using complementary assays, each interrogating different aspects of radical scavenging chemistry. DPPH IC_50_ values across published studies range 0.3 to 0.9 mg/mL for aqueous and methanolic extracts, with *S. herbacea* consistently outperforming other congeners (Figure 2) [17,18]. FRAP values of 50 to 120 μmol Fe^2+^ equivalent/g dw have been reported across species, comparable to values documented for rosemary (*Rosmarinus officinalis*) and significantly exceeding those of common culinary vegetables such as tomato, carrot, and cucumber [9,17]. A systematic comparison by Rodrigues et al. evaluated both *S. ramosissima* and *Sarcocornia perennis* (a related halophyte) under standardised conditions, confirming potent radical scavenging across multiple assay systems with strong linear correlation between TPC and antioxidant activity (r > 0.90), establishing phenolic content as the primary determinant of *Salicornia* antioxidant capacity [17].

The dual-phase antioxidant coverage of *Salicornia* extracts—aqueous-phase protection from hydrophilic phenolic acids and flavonoids, and lipid-phase protection from carotenoids and vitamin E analogues—is a practical technological advantage for food applications where both water-phase and lipid-phase oxidation must be controlled simultaneously. Chlorogenic acid, rutin, and caffeic acid dominate free radical scavenging in aqueous extracts, while the carotenoid fraction (*β*-carotene, lutein, zeaxanthin; 0.1 to 0.5 mg/g dw) provides complementary lipid-phase antioxidant activity [9,18]. This combination is particularly relevant for shelf-life extension applications in fat-containing food matrices, such as emulsified plant-based meat products, where oxidative rancidity represents both a quality and safety challenge. The potential use of *Salicornia* extracts to reduce reliance on synthetic antioxidants such as BHA, BHT, or TBHQ in such formulations represents an underexplored area with clear clean-label relevance [18].

Processing conditions significantly affect the antioxidant capacity of *Salicornia* ingredients. Blanching (85–95 °C, 2–5 min) typically reduces TPC by 15 to 30% through leaching of water-soluble phenolics and thermal degradation of thermolabile compounds [35,36]. High-pressure processing (HPP) has been reported to preserve or marginally enhance TPC relative to thermal treatments in halophyte matrices, likely through cell wall disruption improving polyphenol extractability without thermal degradation [35]. Spray drying and freeze drying both preserve antioxidant capacity more effectively than conventional oven drying, with freeze-dried *Salicornia* powder retaining approximately 80 to 90% of fresh TPC compared with 60 to 70% for spray-dried product [9,18]. These processing-activity relationships are critical for formulation design in functional food applications where the ingredient undergoes heat treatment during production.

## 6. Cardiovascular and Cardiometabolic Effects

**Figure 3:**
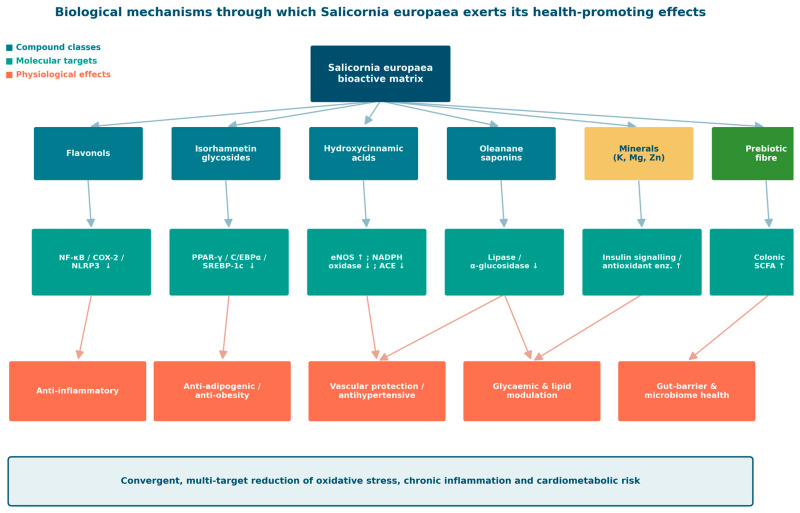
Biological mechanisms through which *Salicornia europaea* exerts its health-promoting effects. Compound classes (blue) act on molecular targets (teal) that translate into physiological effects (orange), converging on a multi-target reduction in oxidative stress, chronic inflammation and cardiometabolic risk. NF-κB = nuclear factor kappa-light-chain enhancer of activated B cells; COX-2 = cyclooxygenase-2; NLRP3 = NLR family pyrin domain containing 3; PPAR-γ = peroxisome proliferator-activated receptor gamma; SREBP-1c = sterol regulatory element-binding protein 1c; eNOS = endothelial nitric oxide synthase; ACE = angiotensin-converting enzyme; SCFA = short-chain fatty acids. Arrows denote the direction of mechanistic influence linking compound classes, molecular targets and physiological effects; upward (↑) and downward (↓) symbols indicate up- or down-regulation (increase or inhibition) of the corresponding target or pathway.

Cardiovascular disease remains the leading cause of global mortality, responsible for an estimated 17.9 million deaths annually according to the WHO 2022 report, with dietary sodium excess, endothelial dysfunction, dyslipidaemia, and chronic low-grade systemic inflammation as primary modifiable pathological drivers. The bioactive profile of *S. europaea* converges mechanistically on multiple cardiovascular targets simultaneously, providing a multilayer rationale for its role as a functional cardioprotective ingredient reflecting multiple complementary bioactive pathways. The mechanistic breadth is illustrated by the convergence of at least four distinct molecular targets: the eNOS/NO vascular protection axis (ferulic acid), the LDL oxidation and endothelial inflammation axis (quercetin, kaempferol), the lipid absorption and hepatic cholesterol synthesis axis (saponins, prebiotic fibre), and the systemic inflammation axis (flavonoids, phenolic acids via NF-κB) [16,22,29,30]. Table 2 summarises the principal preclinical evidence for cardiovascular and cardiometabolic effects. These convergent molecular mechanisms are summarised schematically in Figure 3.

### 6.1. Vascular Protection and the Ferulic Acid–Nitric Oxide Axis

The pivotal study by Kim et al. published in the International Journal of Molecular Sciences provided mechanistic evidence for *Salicornia*’s cardiovascular distinctiveness from crystalline NaCl [16]. Using isolated rat aortic ring preparations and a hypertensive rat model, the investigators demonstrated that *S. europaea* aqueous extract at equivalent sodium concentrations to NaCl failed to induce the endothelium-dependent relaxation impairment, reduced eNOS phosphorylation, and elevated superoxide production characteristic of high-NaCl vascular challenge. Pharmacological fractionation of the extract identified *trans*-ferulic acid as the principal vascular-protective mediator, operating through selective phosphorylation of eNOS at the activating Ser1177 residue, enhanced endothelial NO production, and inhibition of NADPH oxidase-derived superoxide generation, which would otherwise rapidly degrade NO through formation of peroxynitrite [16,22]. The in vivo arm of the study documented a reduction of approximately 15 mmHg in systolic blood pressure in salt-loaded spontaneously hypertensive rats treated with *S. europaea* extract compared with NaCl control at matched sodium dosing—a magnitude of reduction clinically relevant at the individual level and epidemiologically substantial when translated to population exposure [16].

The broader cardiovascular significance of dietary ferulic acid has been established in multiple experimental and epidemiological contexts. Ferulic acid and its principal microbial metabolite dihydroferulic acid, generated by colonic bacterial dehydration and reduction in chlorogenic acid and hydroxycinnamate precursors, inhibit ACE in vitro with Ki values in the low micromolar range, suggesting a pharmacologically plausible contribution to blood pressure regulation through the renin–angiotensin–aldosterone system [22,23]. Ferulic acid has additionally been shown to suppress platelet aggregation through inhibition of thromboxane A2 synthesis, attenuate endothelin-1-mediated vasoconstriction, and reduce monocyte adhesion to activated endothelium through downregulation of VCAM-1 and E-selectin expression, collectively supporting an anti-atherosclerotic profile beyond blood pressure modulation [22]. These mechanisms are relevant not only to hypertension management but to the broader continuum of cardiometabolic risk reduction in the context of metabolic syndrome and T2DM comorbidity.

The potassium content of *S. europaea* (800 to 1200 mg/100 g dw) provides a complementary and independently validated cardiovascular mechanism. Dietary potassium exerts antihypertensive effects through multiple pathways: natriuresis (increasing urinary sodium excretion via competitive tubular reabsorption); vascular smooth muscle relaxation through hyperpolarisation of cell membranes (K^+^/ATPase activation); endothelial protection through suppression of superoxide generation; and potential direct vasodilatory effects on mesenteric and renal vasculature [38,39]. A meta-analysis of 32 randomised controlled trials by Whelton et al. involving 2609 participants found that potassium supplementation reduced systolic blood pressure by a mean of 3.1 mmHg (95% CI: 2.0–4.2) and diastolic blood pressure by 2.0 mmHg, with effect sizes approximately doubling in hypertensive subjects [40]. The systematic review by Aburto et al. further confirmed that higher dietary potassium intake was associated with a 24% lower risk of stroke and a trend toward lower CVD risk across 11 cohort studies [39]. The combined delivery of potassium and reduced-bioavailability sodium in *S. europaea* thus addresses both sides of the electrolyte balance equation implicated in cardiovascular risk.

### 6.2. Lipid Metabolism, LDL Oxidation, and Dyslipidaemia

Atherosclerosis initiation requires the accumulation and oxidative modification of low-density lipoprotein (LDL) within the subendothelial space, creating foam cells through scavenger receptor-mediated uptake in macrophages. The inhibition of LDL oxidation by dietary polyphenols is accordingly a mechanistically proximal anti-atherosclerotic target. Quercetin, kaempferol, and isorhamnetin present in *S. europaea* have individually demonstrated capacity to reduce LDL oxidation in cell-free and cell-based assays through both direct radical scavenging (particularly inhibition of copper-catalysed LDL oxidation, IC_50_ typically 5 to 20 μM for quercetin) and indirect mechanisms including upregulation of paraoxonase-1 (PON-1), the HDL-associated antioxidant enzyme that hydrolyses both arylesterase substrates and lipid peroxides [29,42]. PON-1 upregulation by quercetin has been demonstrated in multiple cell lines and in animal feeding studies, with transcriptional upregulation through Sp1 and HNF-1α binding to the PON-1 promoter region—a mechanism providing durable rather than transient antioxidant protection [29].

Saponins from the oleanane triterpenoid fraction of *Salicornia* exhibit bile acid binding capacity in colonic model systems, reducing the micellar solubilisation of cholesterol in the intestinal lumen and increasing faecal sterol excretion—a mechanism analogous to that of soluble viscous fibres (psyllium, *β*-glucan) and plant sterols/stanols in their cholesterol-lowering capacity [24]. The prebiotic fibre fraction contributes an additional lipid-lowering mechanism through SCFA-mediated inhibition of hepatic de novo cholesterol synthesis: propionate generated by colonic fermentation of soluble *Salicornia* polysaccharides inhibits hydroxymethylglutaryl-CoA (HMG-CoA) reductase, the rate-limiting enzyme of the mevalonate pathway targeted by statin drugs, at the transcriptional level through downregulation of SREBP-2 [26,27,41]. These complementary mechanisms—polyphenol-mediated LDL oxidation inhibition, saponin-mediated bile acid binding, and SCFA-mediated HMG-CoA reductase suppression—collectively constitute a multi-point functional architecture for dyslipidaemia management that is mechanistically distinct from single-target pharmacological intervention.

### 6.3. Anti-Inflammatory Pathways: NF-κB, COX-2, and Systemic Inflammation

Chronic low-grade systemic inflammation is a central pathophysiological feature shared by CVD, T2DM, obesity, and metabolic syndrome, and mediates cross-talk between these conditions through shared molecular effectors: NF-κB, TNF-α, IL-6, IL-1β, MCP-1, CRP, and related inflammatory markers [30]. Flavonoids and phenolic acids in *Salicornia* exert anti-inflammatory activity through NF-κB and AP-1 transcription factor inhibition, operating primarily at the level of IκB kinase (IKKβ) inhibition, preventing phosphorylation and ubiquitin-mediated degradation of the inhibitory IκB proteins that sequester NF-κB dimers in the cytoplasm. The consequence is reduced nuclear translocation of NF-κB p65/p50 heterodimers and suppressed transcription of pro-inflammatory target genes including iNOS, COX-2, TNF-α, IL-6, and adhesion molecules [17,18,30,43]. In LPS-stimulated RAW 264.7 macrophage models, polyphenolic *S. herbacea* extracts produced significant reductions in TNF-α and IL-6 production (*p* < 0.05 vs. LPS control) at extract concentrations of 50 to 200 μg/mL, with quercetin and luteolin identified as the most active individual constituents [17,18].

Prostaglandin E2 (PGE2) and leukotriene B4 (LTB4), inflammatory lipid mediators generated by COX-2 and 5-lipoxygenase respectively, are suppressed by kaempferol and luteolin in activated mast cells and macrophages at concentrations consistent with nutritional exposure, providing complementary eicosanoid-mediated anti-inflammatory activity to the transcription factor-level effects [29,31]. Ferulic acid further contributes through Nrf2/HO-1 pathway activation, upregulating haem oxygenase-1 and NADPH quinone oxidoreductase as endogenous anti-inflammatory and cytoprotective enzymes, a mechanism that also contributes to the ferulic acid-mediated vascular protection described above [22]. The systemic anti-inflammatory activity of *Salicornia*’s polyphenol complex is thus multi-mechanistic and likely synergistic, consistent with the paradigm that complex food matrices exhibit greater biological activity than equivalent doses of isolated individual compounds—an observation consistently supported across polyphenol nutritional pharmacology [28,35,36].

## 7. Metabolic Health, Anti-Obesity Mechanisms, and Glycaemic Modulation

*S. europaea* presents a multi-target preclinical profile for metabolic health and obesity prevention, integrating anti-adipogenic, lipase-inhibitory, glycaemic-modulating, and hepatoprotective mechanisms that are biochemically distinct and potentially synergistic in the context of the metabolic syndrome phenotype. Metabolic syndrome—defined by the joint IDF/AHA/NHLBI consensus statement as the constellation of abdominal obesity, elevated fasting glucose, hypertriglyceridaemia, low HDL-cholesterol, and hypertension [34]—affects approximately 25 to 30% of adults globally by current diagnostic criteria and represents the primary modifiable precursor of T2DM and CVD in the middle-aged population [33,34]. The biological targets addressed by *Salicornia* bioactives—PPAR-γ-mediated adipogenesis, pancreatic lipase-dependent fat absorption, postprandial *α*-glucosidase activity, and hepatic de novo lipogenesis—collectively map onto the metabolic syndrome phenotype, suggesting a plausible functional food positioning for metabolic risk management.

### 7.1. Anti-Adipogenic Activity: Isorhamnetin, PPAR-γ, and the Adipogenesis Cascade

The most mechanistically characterised anti-obesity activity of *Salicornia* is the inhibition of adipocyte differentiation by isorhamnetin-3-*O*-glucoside, established by Park et al. [15]—the seminal in vitro study that first isolated isorhamnetin-3-O-β-D-glucoside from *Salicornia herbacea* and identified it as the principal anti-adipogenic constituent—using the 3T3-L1 preadipocyte model. The dose–response characteristics of this inhibition are dose-dependent: a 40 to 60% reduction in Oil Red O-stained lipid droplet accumulation at 10 to 50 μM isorhamnetin-3-*O*-glucoside, with IC_50_ approximately 20 to 30 μM, achievable in the portal circulation following ingestion of *Salicornia*-enriched food products at realistic serving sizes [15,35]. The molecular cascade suppressed by isorhamnetin encompasses the complete transcriptional programme of adipogenesis: early-phase C/EBPβ and C/EBPδ induction is attenuated, preventing activation of the mid-phase PPAR-γ and C/EBPα master regulators, which in turn suppresses the late-phase differentiation programme including FABP4/aP2, perilipin-1, glycerol-3-phosphate dehydrogenase (GPDH), and lipoprotein lipase (LPL) expression [15,32].

This anti-adipogenic mechanism is of particular clinical relevance in the context of visceral adiposity, the adipose depot most pathogenically linked to insulin resistance, atherogenic dyslipidaemia, and pro-inflammatory cytokine secretion (adipokine dysregulation) in metabolic syndrome [33,34]. Visceral adipocyte hypertrophy and hyperplasia drive elevated circulating FFA, TNF-α, IL-6, and resistin concentrations while reducing adiponectin—an adipokine with insulin-sensitising and anti-inflammatory properties whose circulating levels are inversely correlated with cardiometabolic risk. The PPAR-γ inhibitory activity of isorhamnetin glycosides from *Salicornia* operates upstream of this adipokine dysregulation cascade, and may represent a preventive strategy for metabolic syndrome incidence in nutritional risk management. Complementary to the direct anti-adipogenic effect, isorhamnetin has been shown by Lv et al. to attenuate hepatic steatosis in high-fat diet mouse models through inhibition of SREBP-1c-driven de novo lipogenesis and reduction in hepatic oxidative stress, suggesting relevance to the spectrum of non-alcoholic fatty liver disease (NAFLD)—the hepatic manifestation of metabolic syndrome—which affects an estimated 25% of the global adult population [21].

### 7.2. Enzyme Inhibition: Pancreatic Lipase and α-Glucosidase

Saponin-enriched fractions of *Salicornia* inhibit porcine pancreatic lipase (PPL) in vitro, with IC_50_ values of approximately 0.3 mg/mL reported in enzyme kinetics assays [25]. Pancreatic lipase catalyses the primary step in dietary triglyceride digestion—hydrolysis to monoglycerides and fatty acids at the oil-water interface of lipid emulsions—and its inhibition by orlistat (IC_50_ ~0.04 μg/mL) is the mechanistic basis of the only approved pharmacological anti-obesity drug targeting fat absorption. While *Salicornia* saponins are less potent than orlistat, they operate without the adverse gastrointestinal effects (steatorrhoea, faecal urgency, fat-soluble vitamin malabsorption) associated with pharmacological lipase inhibition at therapeutic doses, and are more compatible with food-grade application. Kinetic analysis suggests competitive-type inhibition, with the glycoside chain mediating interaction with the lipase active site through hydrophobic and hydrogen-bonding interactions [24,25]. Consistent with this enzyme-directed activity, *Salicornia brachiata* seed oil rich in linoleic acid has recently been shown to inhibit α-glucosidase and to lower blood glucose and serum lipids in mice, reinforcing the antidiabetic and anti-obesity relevance of the genus [44].

Quercetin and isorhamnetin aglycones additionally demonstrate *α*-glucosidase inhibitory activity in vitro, attenuating postprandial glucose excursions by slowing intestinal starch hydrolysis and reducing the rate of glucose monomers available for intestinal absorption. IC_50_ values for quercetin against sucrase-isomaltase complex are reported in the range 0.15 to 0.4 mg/mL in multiple independent studies, representing modest but potentially clinically meaningful inhibition in the context of sustained dietary exposure [29,42]. The combination of soluble fibre-mediated gastric emptying delay, phenolic acid-mediated inhibition of intestinal glucose transporters SGLT1 and GLUT2 (documented for chlorogenic acid at physiologically relevant concentrations), and flavonol-mediated *α*-glucosidase inhibition creates a multi-mechanistic glycaemic modulation platform within a single *Salicornia* ingredient—mechanistically distinct from single-compound interventions and potentially more robust against adaptive resistance. This functional profile is mechanistically relevant to dietary strategies targeting postprandial glycaemic control, although its clinical significance in prediabetic and T2DM populations requires direct human intervention evidence [22,33,42].

### 7.3. Magnesium, Zinc, and Insulin Signalling

The high magnesium content of *S. europaea* (200 to 400 mg/100 g dw) has additional mechanistic relevance to insulin action and metabolic health that is independent of its antihypertensive cardiovascular activity. Magnesium is an essential cofactor for over 300 enzymatic reactions, including pyruvate dehydrogenase, all glycolytic kinases, and the insulin receptor tyrosine kinase [33,34]. Magnesium deficiency—prevalent in populations consuming high-sodium, processed-food diets—is associated with impaired insulin signalling through reduced insulin receptor substrate-1 (IRS-1) phosphorylation, elevated intracellular calcium (competing with Mg for binding sites on signalling proteins), and increased oxidative stress [33]. Epidemiological data from large cohort studies, including the PREDIMED trial and several European population studies, have consistently shown inverse associations between dietary magnesium intake and T2DM incidence, independent of other dietary variables [34,45]. The zinc content of *S. europaea* (2 to 5 mg/100 g dw) contributes further to insulin biology: zinc is essential for proinsulin processing to insulin in pancreatic beta cells (zinc co-crystallises with insulin in secretory granules), for insulin receptor signalling, and for antioxidant protection of beta cell mitochondria through metallothionein and copper-zinc SOD pathways [33,34]. In the context of plant-based diets, where zinc bioavailability is constrained by phytate content in grains and legumes, *Salicornia*’s contribution of mineral zinc in a relatively low-phytate matrix is nutritionally significant.

## 8. Gut Microbiome Modulation and Intestinal Health

The gut microbiome—the collective genome and metabolic activity of the approximately 10^13^ microorganisms inhabiting the human gastrointestinal tract—has been firmly established as a critical mediator of host nutrition, immune function, and metabolic homeostasis. Previous research has established the bidirectional interaction between dietary composition and microbiome structure, demonstrating that changes in macronutrient composition can remodel the gut microbial community within days and that microbiome composition in turn determines host energy extraction and metabolic outputs from ingested food [46]. The relationship between dietary fibre and gut microbiome health is particularly well characterised: fermentable polysaccharides selectively stimulate health-promoting commensal bacteria (*Bifidobacterium* spp., *Lactobacillus* spp., *Roseburia* spp., Faecalibacterium prausnitzii) whose metabolic products—primarily short-chain fatty acids—exert direct effects on host metabolism, immune regulation, and intestinal barrier integrity [26,27].

*Salicornia* aerial parts contain 15 to 25 g/100 g dw of total dietary fibre, with the soluble fermentable fraction (25 to 30%) constituting the principal prebiotic substrate. Ex vivo faecal fermentation studies using human donor microbiota have demonstrated that *Salicornia* polysaccharide fractions selectively increase *Bifidobacterium* and *Lactobacillus* enumeration with prebiotic index (PI) values comparable to inulin at equivalent substrate concentrations [26,27]. This selectivity reflects the specific glycosidic linkages and branching patterns of the galacturonan, mannan, and arabinoxylan fractions, which are fermentable only by bacteria possessing the appropriate carbohydrate-active enzymes (CAZymes)—a molecular precision that underpins the prebiotic selectivity concept [26]. SCFA production in these fermentation models includes butyrate (20 to 30% of total SCFA), propionate (25 to 35%), and acetate (35 to 50%), consistent with a mixed substrate fermentation profile supporting colonocyte energy supply (butyrate), hepatic gluconeogenesis regulation (propionate), and peripheral energy metabolism (acetate) [27,41]. In line with this prebiotic profile, *Salicornia*-derived xylo-oligosaccharides have themselves been characterised as prebiotic substrates that increase gut microbial diversity and enrich beneficial symbionts [47].

Butyrate deserves particular attention as a functional output of *Salicornia* fibre fermentation. As the primary energy substrate of colonocytes and an endogenous histone deacetylase (HDAC) inhibitor, butyrate plays a dual role in maintaining intestinal barrier integrity (upregulating tight junction proteins claudin-1, occludin, and ZO-1) and regulating mucosal immune homeostasis through suppression of NF-κB in colonocytes and induction of regulatory T cells (Treg) in intestinal mucosa [27,41]. Impaired intestinal barrier integrity—manifested as increased paracellular permeability (“leaky gut”)—is a documented feature of obesity, T2DM, and metabolic syndrome, associated with translocation of bacterial lipopolysaccharide (LPS) into the systemic circulation (metabolic endotoxaemia) and subsequent TLR4-mediated inflammatory cascade activation in adipose tissue and liver. Dietary strategies that enhance butyrate production and intestinal barrier function thus address a mechanistically upstream driver of metabolic inflammation, and *Salicornia*’s dual prebiotic fibre and anti-inflammatory polyphenol profile positions it as a potentially synergistic modulator of this gut-systemic inflammation axis [26,27,41,46].

The polyphenol fraction of *Salicornia* contributes additionally to gut health through a complementary mechanism: colonic microbial biotransformation of flavonoids and phenolic acids generates bioavailable, structurally distinct metabolites with enhanced systemic bioactivity compared with the parent compounds [35,36]. Quercetin glycosides undergo intestinal deglycosylation and colonic ring-fission to yield phenylpropionic acids (3,4-dihydroxyphenylpropionic acid, 3-hydroxyphenylpropionic acid), which are absorbed and exert systemic anti-inflammatory effects; ferulic acid and chlorogenic acid are converted to dihydroferulic acid and caffeic acid derivatives by colonic reductases, enhancing bioavailability and potentially extending anti-inflammatory activity [35,36]. This biotransformation creates a positive functional feedback: the prebiotic fibre sustains the specific microbial populations (*Lactobacillaceae*, *Bifidobacteriaceae*, *Lachnospiraceae*) possessing the polyphenol-metabolising enzymes, while the polyphenols selectively modulate gut microbiome composition in favour of health-promoting taxa through their bacteriostatic effects on pathogenic Gram-negative bacteria—an integrated polyphenol-prebiotic synergy unique to complex food matrices [26,46].

## 9. Antimicrobial and Additional Bioactivities

Beyond the cardiovascular, metabolic, and gut microbiome effects that constitute the primary pharmacological axes of functional interest, *Salicornia europaea* extracts exhibit additional biological activities that are mechanistically documented, albeit explored in less depth in the current literature. Antimicrobial activity against a range of food-relevant and clinically relevant pathogens has been reported for both aqueous and organic solvent extracts. Minimum inhibitory concentrations (MIC) of 0.5 to 2 mg/mL have been documented for methanolic *S. europaea* extracts against *Staphylococcus aureus*, *Listeria monocytogenes*, and *Salmonella typhimurium* in disc diffusion and broth microdilution assays, with moderate activity against *Escherichia coli* [9,18]. The antimicrobial mechanism is attributable primarily to the flavonoid fraction: quercetin disrupts membrane integrity in Gram-positive bacteria through interaction with lipoteichoic acid and membrane phospholipids; caffeic acid inhibits cell wall synthesis; and combined polyphenol-mineral (zinc) activity provides synergistic bacteriostatic effects against *S. aureus* [18]. These antimicrobial properties may be relevant to food preservation applications, where *Salicornia* extracts could be explored as natural preservative adjuncts for minimally processed foods—a clean-label application aligned with contemporary food industry trends away from synthetic preservatives such as sodium benzoate, potassium sorbate, and nisin [9].

Preliminary evidence also supports tyrosinase inhibitory activity of *Salicornia* polyphenol extracts, with IC_50_ values in the range 0.5 to 1.5 mg/mL reported for caffeic acid and quercetin fractions [9]. Tyrosinase catalyses the rate-limiting steps in melanin biosynthesis and is the primary target for skin-lightening cosmeceutical applications. While this activity falls outside the nutritional scope of the present review, it flags the potential of *Salicornia* extracts in dermocosmetic functional applications—an additional translational application of the plant’s phytochemical profile. Acetylcholinesterase (AChE) inhibitory activity has been detected in crude polyphenol fractions of related halophytes at concentrations relevant to topical or nutraceutical applications [9], though systematic characterisation of this activity specifically in *S. europaea* at nutritionally achievable concentrations has not been published, representing a research gap with potential relevance to cognitive health positioning in ageing population segments.

### Antiproliferative and Anti-Cancer Activity

Emerging evidence indicates that *Salicornia* extracts and their constituents possess antiproliferative and anti-cancer activity—an axis only recently explored for this genus and consistent with the broader anticancer potential attributed to marine-derived bioactives [12]. In an in vivo model of colitis-associated colorectal carcinogenesis, dietary supplementation with ground *Salicornia herbacea* suppressed azoxymethane/dextran-sulphate-sodium (AOM/DSS)-induced colon tumour formation in mice, an effect mechanistically linked to downregulation of Wnt/β-catenin signalling and modulation of the Nrf2 cytoprotective pathway [48]. At the macromolecular level, a structurally characterised polysaccharide isolated from the halophyte *Salicornia bigelovii* (SabPS-1) inhibited the proliferation of HepG2 hepatocarcinoma cells in vitro and induced apoptosis through caspase-3/-8 activation and an increased Bax/Bcl-2 ratio [49]. Complementary in silico work has shown that the principal *Salicornia* flavonoids—isorhamnetin, quercetin, myricetin, and kaempferol—together with caffeoylquinic acids exhibit favourable drug-likeness and binding affinity toward members of the human epidermal growth factor receptor (HER) family implicated in carcinogenesis, providing a molecular rationale for the observed antiproliferative effects [50]. Importantly, several mechanisms already described in this review—NF-κB suppression, Nrf2/HO-1 activation, and attenuation of chronic oxidative and inflammatory signalling—are themselves recognised anti-carcinogenic pathways, suggesting that the anti-cancer potential of *Salicornia* is mechanistically continuous with its antioxidant and anti-inflammatory activities rather than a separate property [12,48,49]. This evidence nonetheless remains preclinical and, in part, computational; dose–response characterisation and in vivo validation specific to *S. europaea* are required before any oncological positioning can be advanced.

## 10. Functional Food Applications in Precision Nutrition

The convergence of regulatory compatibility, organoleptic acceptability, multi-target bioactivity, and sustainable agronomy supports the positioning of *Salicornia europaea* as a versatile marine-derived functional ingredient with potential relevance to future precision nutrition applications. Its implementation across diverse food matrices—from condiments and electrolyte beverages to plant-based proteins and prebiotic preparations—reflects the technological breadth enabled by its aqueous solubility, heat stability of the mineral fraction, and the dose relevance of bioactives at reasonable serving sizes. Table 3 summarises the principal functional food application matrices, with corresponding technological considerations and health positioning.

### 10.1. Sodium Reduction and Functional Electrolyte Delivery

*Salicornia*-derived salt—typically produced as a fine or coarse crystalline powder from whole-plant spray-dried or oven-dried ground biomass, or as evaporated brine concentrate from washed plant material—provides 40 to 50% less bioavailable sodium per gram than an equivalent mass of crystalline NaCl while delivering a complementary marine mineral matrix of potassium, magnesium, and calcium, which is consistent with the mineral profile considered favourable in the context of sodium-excess Western dietary patterns [16,38]. Unlike KCl-based salt substitutes, which introduce a bitter metallic taste note perceptible at concentrations above 20 to 30% replacement and typically require flavour-masking interventions, *Salicornia* salt exhibits a mild, slightly marine-umami organoleptic character at functional doses (0.5 to 2 g per serving)—attributable to its naturally elevated glutamate and betaine content—that is compatible with diverse food matrices without perceptible off-flavour [9,51]. Sensory panel studies in European markets have confirmed consumer acceptance of *Salicornia*-seasoned products relative to equivalent NaCl and mixed KCl/NaCl blends, with preference scores favouring *Salicornia* in food contexts where provenance and natural origin are primary purchasing drivers [51].

The regulatory and claims infrastructure for *Salicornia*-based sodium reduction is highly favourable in the EU context. Commission Regulation (EU) 432/2012 authorises the health claim that potassium contributes to the maintenance of normal blood pressure for foods that qualify at least as a source of potassium. Under EU nutrient reference values, potassium has an NRV of 2000 mg; therefore, the usual ‘source of potassium’ threshold corresponds to 300 mg per 100 g for foods other than beverages, while ‘high potassium’ requires at least twice that amount [52]. These thresholds are achievable in dry seasoning and condiment products incorporating *Salicornia* powder at 10 to 20% inclusion rates, which also enable the associated authorised claim that “potassium contributes to normal blood pressure maintenance.” Magnesium claims (“contributes to normal energy-yielding metabolism,” “contributes to normal muscle function,” “contributes to normal protein synthesis”) are similarly achievable, providing a multi-claim regulatory framework not commonly available from a single plant-derived ingredient under current EU regulation [52,53]. The possibility of supporting several EFSA-authorised nutrient content claims from a single marine ingredient is therefore relevant to the design of functional food applications.

Sodium reduction in processed foods is a broader reformulation challenge beyond seasoning. The SSaSS trial—demonstrating a 14% stroke reduction from substituting 25% of NaCl with KCl in a rural Chinese population—provides population-level evidence supporting salt substitution as a public health strategy [7]. The WHO SHAKE technical package explicitly endorses salt substitution as a component of comprehensive sodium reduction policy, and multiple national food agencies have implemented mandatory or voluntary sodium reduction targets that create incentives for ingredient reformulation [51]. In this policy context, *Salicornia*-based sodium reduction ingredients are positioned not merely as functional food options but as technically viable complements to population-wide dietary sodium reduction programmes—particularly in bakery, processed meat, dairy, and convenience food categories where flavour-matching with NaCl has historically constrained the adoption of salt substitutes [6,51].

### 10.2. Plant-Based Meat and Protein Applications

Plant-based meat alternatives represent a relevant application context for *Salicornia* as a multi-functional ingredient. In plant-based burger, sausage, and nugget formulations, protein texturisation processes (high-moisture extrusion, wet texturisation of soy or pea protein concentrate) generate neutral or mildly earthy flavour profiles that benefit from savoury seasoning without the sodium burden of conventional salt-based seasoning blends. *Salicornia* powder at 0.5 to 2% inclusion serves simultaneously as a sodium-reduced savoury flavour note (umami from betaine, glutamate, and mineral electrolytes), a magnesium and zinc fortification source addressing nutritional gaps commonly documented in plant-based diets (notably zinc bioavailability limitations from grain and legume-based protein sources), and a prebiotic fibre contributor enhancing the overall dietary fibre content of the product [8,9]. The clean-label botanical origin of *Salicornia* is compatible with USDA Organic, EU Organic, and Non-GMO Project verification pathways, providing supply chain documentation advantages compared with mineral fortification using synthetic mineral salts.

### 10.3. Functional Beverages and Hydration Products

In functional clear-beverage applications—isotonic sports drinks, electrolyte waters, wellness shots, and recovery beverages—the principal challenge for *Salicornia* incorporation is achieving functional electrolyte doses without exceeding sensory threshold concentrations that would compromise taste neutrality in clear liquid matrices. The sensory threshold for *Salicornia* extract in water has been characterised at approximately 0.8 to 1.2 g/L for most consumer panels, above which a characteristic mild marine note becomes perceptible—a threshold that comfortably allows incorporation at electrolyte-functional doses (providing 50 to 150 mg potassium and 20 to 50 mg magnesium per serving) without organoleptic compromise [9]. This positions *Salicornia*-derived electrolytes as technically viable alternatives to synthetic mineral blends (potassium citrate, magnesium sulphate, sodium chloride) in functional hydration products, with the added differentiation of natural marine origin, clean label, and co-delivery of polyphenol microdoses. This applied profile is consistent with *Salicornia*’s ingredient profile [8].

### 10.4. Sustainability and Circular Economy Positioning

*Salicornia* cultivation represents a paradigmatic circular economy proposition with multiple dimensions relevant to contemporary ESG-aligned food industry strategy. Agronomically, the plant utilises coastal and inland saline lands that are marginalised for conventional food production—estimated at 250 to 300 million hectares globally [8]—without displacing food crops from arable land. Irrigation with brackish groundwater or treated wastewater conserves freshwater resources and can rehabilitate salt-affected soils over successive growing cycles. Minimal agrochemical inputs—achievable owing to the extreme salinity tolerance that competitively excludes most pathogens and weeds from the growing environment—reduce both production costs and environmental contamination loads. Life cycle assessment (LCA) data from Mediterranean *Salicornia* production systems indicate global warming potentials of 0.8 to 1.2 kg CO2-equivalent per kg of dry biomass, compared with 1.5 to 2.5 kg CO2-equivalent for conventional mineral salt production, and substantially lower than potassium chloride extraction (3 to 5 kg CO2-equivalent/kg) [8]. The by-product stream of *Salicornia* salt production—the fibrous press cake after brine extraction—retains the full polyphenol, fibre, and mineral complement of the original biomass and can be valorised as a functional food ingredient in its own right, creating a zero-waste circular value chain.

### 10.5. Documented Functional-Food and Translational Evidence

Although several of the applications discussed above remain prospective, a growing body of recent work has begun to document the use of *Salicornia* in actual food and nutraceutical contexts, partly addressing the long-standing scarcity of applied evidence noted by previous authors. At the clinical end, a randomised, triple-blind, placebo-controlled trial administered a polyphenol-rich *Salicornia* extract to patients recovering from transient ischaemic attack or minor stroke and reported reductions in plasma homocysteine and blood pressure, providing the first controlled human evidence that a *Salicornia*-based functional ingredient can favourably modulate cardiovascular-risk markers [54]. At the ingredient-characterisation level, comparative chemical fingerprinting of *S. europaea* and related species has confirmed reproducible antioxidant and antidiabetic (α-amylase, α-glucosidase, and SGLT1-related) activities relevant to functional-food formulation [55], while green, NADES-based ultrasound-assisted extraction of Apulian *S. europaea* has been optimised to recover isorhamnetin-rich fractions with antioxidant and antibacterial activity suitable as natural food additives [56]. Simulated gastrointestinal-digestion and intestinal-permeability models of *Salicornia* by-products have demonstrated that the principal caffeoylquinic acids remain bioaccessible and able to cross the intestinal barrier, supporting their valorisation as nutraceutical ingredients within a circular-economy framework [57]. From a product-technology standpoint, controlled-atmosphere and temperature studies of hydroponically grown glasswort have defined the post-harvest conditions that preserve its biochemical and sensory quality during storage—a prerequisite for its use as a fresh functional vegetable [58]—and ethyl-acetate fractions of *S. europaea* have been shown to induce adipocyte browning in vitro, extending the documented anti-obesity functionality of the ingredient [59]. Taken together, these studies move *Salicornia* from a theoretically promising matrix toward an evidence-supported functional-food ingredient, even though the clinical literature remains small and is currently dominated by a single cardiovascular trial.

## 11. Bioavailability, Processing Effects, and Matrix Interactions

The systemic bioavailability of phytochemicals from food matrices is a critical determinant of their in vivo pharmacological activity and cannot be inferred from in vitro biological effects measured at extract concentrations far exceeding those achievable in plasma or target tissues. For *Salicornia* polyphenols, bioavailability follows the general pharmacokinetic framework established for dietary flavonoids, with important matrix-specific modulations. Quercetin glycosides (principally the glucoside form, isoquercitrin) are the best-characterised flavonoids in terms of intestinal absorption: deglycosylation by brush border lactase phlorizin hydrolase (LPH) in the small intestine and/or cytosolic glucosidase (CBG) after cellular uptake generates quercetin aglycone, which is transported across the enterocyte membrane by passive diffusion and GLUT2-facilitated transport. Systemic bioavailability of quercetin from food matrices is reported in the range 20 to 50%, substantially higher than that of the rutinoside form (quercetin-3-*O*-rutinoside; bioavailability approximately 5 to 17%) owing to the requirement for colonic microbial *α*-rhamnosidase and *β*-glucosidase activity for rutinoside deglycosylation [35,36,60]. This distinction is relevant to the functional ingredient design question: processing methods that preferentially preserve the glucoside over the rutinoside form of quercetin in *Salicornia* would enhance systemic bioavailability of the absorbed flavonol fraction.

Phenolic acids exhibit generally higher bioavailability than flavonoids. Ferulic acid and caffeic acid, primarily present in ester-bound form within the cell wall matrix of *Salicornia*, are partially liberated by intestinal esterases and partially reach the colon intact where microbial deesterification generates free hydroxycinnamic acids [22,23]. Free ferulic acid is efficiently absorbed in the small intestine with bioavailability estimates of 40 to 70%, while bound ferulic acid from cell wall matrices requires microbial release with variable efficiency dependent on microbiome composition [22]. Chlorogenic acid undergoes sequential hydrolysis by intestinal and microbial esterases to caffeic acid and quinic acid, with overall bioavailability of caffeic acid from chlorogenic acid sources in the range of 15 to 30% [23]. These bioavailability parameters are relevant to dose calculation in functional food design: a *Salicornia* serving providing 5 to 10 mg total ferulic acid equivalent per day would deliver approximately 2 to 7 mg systemically bioavailable ferulic acid—concentrations relevant to the vascular protection mechanisms characterised in vitro and ex vivo [16,22].

Processing and food matrix effects on *Salicornia* bioactive content and stability deserve systematic consideration for functional food product development. Thermal treatment (blanching 85 to 95 °C for 2 to 5 min) reduces water-soluble phenolics by 15 to 30% through leaching and Maillard reaction-associated losses [35,36]. High-pressure processing (HPP; 400 to 600 MPa) and pulsed electric field (PEF) treatment have been reported to preserve or marginally enhance TPC relative to equivalent thermal treatments in halophyte matrices, likely through membrane disruption improving cellular extractability without equivalent thermal degradation [18]. The mineral fraction—potassium, magnesium, calcium, iron, zinc—is fully stable under standard food processing conditions including pasteurisation (72 °C/15 s or 85 °C/30 min), UHT treatment, and HPP, ensuring mineral functionality is preserved irrespective of the thermal process applied [9,16]. Dietary fibre fractions are similarly stable to thermal processing, though viscosity-forming capacity of soluble polysaccharides can be reduced by high-shear mixing and extended thermal treatment. In summary, the mineral and fibre functional attributes of *Salicornia* are process-stable, while the polyphenol fraction requires careful process optimisation to maximise bioactive retention from raw ingredient to finished product.

## 12. International Regulatory Framework and Market Considerations

The international regulatory landscape is highly relevant to the positioning of *S. europaea* as a functional ingredient. Table 4 summarises the regulatory status across six major jurisdictions, indicating that the plant retains traditional food status in most of these contexts and therefore does not fall under Novel Food classification, in contrast to other marine-derived bioactive ingredients (such as fucoxanthin, astaxanthin from non-traditional sources, or coral-derived bioactives) for which dedicated safety dossiers and pre-market approval are typically required.

In the European Union, *S. europaea* (commercially known as samphire or salicornes) carries a long and documented history of consumption as a coastal vegetable across the British Isles, France, and the Netherlands—consumption documented in culinary and ethnobotanical sources dating back to the 16th century. This establishes its status as a traditional food under Regulation (EU) 2015/2283 on novel foods, which explicitly excludes foods with a significant history of safe consumption in the Union from Novel Food classification [53]. The practical consequence is that *Salicornia* powder, extract, or salt can be introduced into EU food products without EFSA safety evaluation, pre-market notification, or Novel Food authorisation—a regulatory pathway that substantially reduces time-to-market compared with the 18 to 36 month dossier preparation and EFSA review timeline applicable to genuinely novel marine ingredients. Health claims based on Commission Regulation (EU) 432/2012 are achievable: potassium contributing to normal blood pressure maintenance (NRV 2000 mg, “source” threshold 300 mg/100 g); magnesium contributing to normal energy-yielding metabolism, muscle function, protein synthesis, and reduction in tiredness; calcium contributing to normal bone and teeth maintenance [52]. These are nutrient-specific, low-threshold claims that require only compositional documentation and appropriate dosing rather than dedicated clinical trial evidence, which is relevant from a regulatory standpoint when developing functional food applications.

South Korea’s regulatory framework around *S. herbacea* (hamcho) represents the most developed evidence-and-regulation nexus for *Salicornia* globally. The Korean Food Standards Codex recognises hamcho as a traditional food ingredient with centuries of documented therapeutic and culinary application in coastal communities of Jeolla and Gyeongnam provinces. The National Institute of Food and Drug Safety Evaluation (NIFDS) has registered standardised *Salicornia herbacea* extracts in the nutraceutical category for glycaemic management indications, supported by the preclinical evidence base reviewed herein. The relative abundance of clinical research on *S. herbacea* in the Korean scientific literature—including placebo-controlled human studies examining glycaemic parameters, liver function markers, and lipid profiles—provides a regulatory and scientific pathway for structured health claim development in the Korean and broader Asia-Pacific market context that is more advanced than that available for *S. europaea* in Western markets. Comparative characterisation of S. herbacea and *S. europaea* bioactive profiles may help assess the extent to which Korean regulatory and clinical precedents are informative for EU and North American health claim development, although species-specific evidence would remain necessary. No reports of serious adverse events, dose-dependent toxicity, or clinically significant drug interactions attributable specifically to *Salicornia* consumption have been documented in the published literature or reported to regulatory agencies in any jurisdiction that has reviewed the ingredient [18,53]. In vitro cytotoxicity studies employing standard human cell lines (HepG2 hepatocytes, Caco-2 enterocytes, RAW 264.7 macrophages) have confirmed absence of significant cytotoxic effects at extract concentrations corresponding to at least 10-fold the concentrations expected from realistic dietary exposure, with LC_50_ values consistently exceeding 500 μg/mL in tested cell systems [17,18]. The primary safety consideration specific to *Salicornia* as a halophytic food ingredient is heavy metal bioaccumulation: plants adapted to high-salinity coastal environments may accumulate trace metals—cadmium, lead, arsenic, mercury—from contaminated coastal or industrial sediments through the same ion transport mechanisms that enable salt tolerance [8,14]. This risk is site-specific rather than intrinsic to the species and is effectively managed through rigorous cultivation site selection, soil and water quality monitoring, and post-harvest washing protocols. EU heavy metal limits for food (Commission Regulation (EU) 2023/915 on maximum levels for certain contaminants in food) provide clear compliance benchmarks against which *Salicornia* ingredient batches must be tested before commercial release [53].

The high potassium content of *Salicornia*-derived salt (approximately 5 to 10% of NRV per gram of product at typical serving concentrations) warrants consideration in individuals with renal impairment (chronic kidney disease stages 3 to 5) who may require dietary potassium restriction due to impaired urinary potassium excretion. This is not a safety concern for the general healthy population—dietary potassium at levels achievable from *Salicornia* food incorporation is consistently associated with health benefit in this group—but should be flagged in product labelling for renal patient populations, analogous to existing labelling conventions for KCl-containing salt substitutes [38,39]. The sodium content, while lower in bioavailability than crystalline NaCl, remains present in the ingredient matrix, and individuals on strict medical sodium restriction (e.g., heart failure NYHA III–IV, severe hypertension under pharmacological management) should account for dietary *Salicornia* in their daily sodium balance calculations, with appropriate guidance from healthcare providers.

## 13. Research Gaps and Future Directions

Despite the compelling preclinical mechanistic evidence reviewed herein, the translational evidence base for *Salicornia europaea* as a functional food ingredient in human health contains critical gaps that currently prevent the development of evidence-based clinical health claims beyond nutrient content claims. These gaps are not peripheral—they represent the primary limiting constraint on the scientific and commercial maturation of *Salicornia* as a mainstream precision nutrition ingredient. Systematic identification and prioritisation of these gaps is necessary for directing research investment toward the studies most likely to yield translational returns.

The most consequential gap is the virtual absence of well-powered human randomised controlled trials (RCTs). A review of the clinical literature reveals that the overwhelming majority of health evidence for *S. europaea* derives from in vitro cell models and rodent in vivo studies, with only observational and traditional use data available for human populations. The one dimension that has partial human analogy—the cardiovascular benefit of salt substitution—is documented through the SSaSS trial [7] and potassium supplementation meta-analyses [40], but these were not conducted with *Salicornia*-specific ingredients and cannot be directly attributed to the compound bioactive profile of this ingredient. A protocol for a prospective, double-blind, crossover RCT examining the acute and chronic (8 to 12 weeks) effects of *Salicornia*-derived salt versus NaCl and KCl/NaCl blend on ambulatory blood pressure, endothelial function (flow-mediated dilation), plasma biomarkers of oxidative stress (F2-isoprostanes, 8-OHdG), and inflammatory markers (hsCRP, IL-6, TNF-α) in prehypertensive adults would represent the highest-priority research need. Secondary endpoints should include 24-h urinary sodium and potassium excretion (to characterise electrolyte bioavailability differences), postprandial lipid response, and microbiome composition assessment by 16S rRNA amplicon sequencing.

Standardisation of *Salicornia* bioactive content across cultivars, growing substrates, harvest timing, and processing conditions is a second critical gap with direct implications for both research reproducibility and commercial product development. Current inter-study variability in reported TPC, flavonol profile, and mineral concentrations is substantial—spanning two to four-fold ranges across published datasets—reflecting a combination of genuine biological variability (substrate salinity effects on secondary metabolite accumulation, phenological stage at harvest, ecotype variation) and methodological heterogeneity (extraction solvent, analytical method, calculation basis) [9,18]. Without agreed Good Agricultural Practice (GAP) standards and validated analytical reference methods for *Salicornia* ingredients, comparative interpretation of study results across research groups and commercial batch-to-batch reproducibility for functional food applications are compromised. Development of a standardised *Salicornia* ingredient monograph—analogous to United States Pharmacopeia (USP), European Pharmacopoeia (EP), or American Herbal Products Association (AHPA) standards for established botanical ingredients—incorporating minimum TPC, total flavonoid content, isorhamnetin-3-*O*-glucoside, and ferulic acid specifications, along with heavy metal limits and microbial counts, is a necessary infrastructure investment for this ingredient category.

The bioavailability and pharmacokinetics of isorhamnetin glycosides from *Salicornia* food matrices in humans have not been characterised. Plasma kinetics studies measuring plasma isorhamnetin, isorhamnetin glucuronide, and isorhamnetin sulphate concentrations after ingestion of standardised *Salicornia*-containing test meals, coupled with urinary metabolite analysis, would establish the systemic exposure achievable from realistic dietary use and enable comparison with the in vitro concentrations at which anti-adipogenic and anti-inflammatory activities have been demonstrated. This pharmacokinetic data is indispensable for substantiating the mechanistic relevance of the in vitro evidence to human physiology—an argument that regulatory bodies, particularly the European Food Safety Authority (EFSA) Panel on Nutrition, Novel Foods, and Food Allergens (NDA), require for health claim substantiation beyond nutrient content claims.

Investigation of the gut microbiome response to *Salicornia*’s dual prebiotic-polyphenol matrix in human subjects is a fourth priority area. Current evidence is extrapolated from ex vivo fermentation models using human faecal inocula—valuable but limited by the inability to capture host-microbiome cross-talk, intestinal transit dynamics, or systemic immune consequences. A human intervention study design incorporating faecal metagenomics (shotgun sequencing at species resolution), faecal metabolomics (targeted SCFA quantification and untargeted metabolite profiling), and plasma polyphenol metabolite measurement would comprehensively characterise the gut microbiome response and the systemic metabolite generation pathway from *Salicornia* polysaccharide and polyphenol substrates. The potential synergy between prebiotic fibre-mediated SCFA production and polyphenol-mediated modulation of gut microbial composition is a particularly interesting mechanistic hypothesis—one that could, if confirmed in humans, differentiate *Salicornia* from single-function prebiotic ingredients [26,27,41,46].

Finally, the effects of projected climate change on *Salicornia* bioactive composition require systematic assessment. Changes in sea level, coastal salinity gradients, temperature, and UV radiation patterns will alter the osmotic and oxidative stress environment that drives secondary metabolite accumulation in this species. Modelling studies suggest that increasing substrate salinity, within the tolerance range of *S. europaea*, may upregulate polyphenol biosynthesis as a stress-adaptive response—potentially enhancing the very bioactives that confer pharmacological value [14,19]. Conversely, extreme salinity or temperature stress may shift metabolic investment from secondary to primary metabolism, reducing polyphenol concentrations. Long-term field studies examining bioactive profiles in natural *Salicornia* populations under contrasting salinity and temperature regimes, combined with controlled growth chamber experiments, would provide the predictive data needed to ensure that future cultivation practices under climate-altered conditions preserve the pharmacological profile that makes this ingredient functionally relevant.

### Limitations of This Review

Several limitations of the present review should be acknowledged. First, this is a narrative rather than a systematic review; although a structured, multi-database search strategy was applied, study selection did not follow PRISMA procedures and was not accompanied by a formal risk-of-bias appraisal or quantitative synthesis, so a degree of selection and interpretation bias cannot be excluded. Second, much of the integrated evidence derives from related congeners (notably *S. herbacea*, *S. ramosissima*, *S. bigelovii*, and *S. brachiata*) rather than from *S. europaea* itself; despite the conserved bioactive architecture of the genus, species-level differences in flavonoid and mineral profiles mean that some inferences are supportive rather than fully substitutive. Third, the mechanistic and efficacy data are overwhelmingly in vitro and in vivo (animal), frequently at concentrations exceeding those plausibly achievable in human plasma, and human clinical evidence remains limited to a single controlled trial and to salt-substitution studies not conducted with *Salicornia*-specific ingredients. Finally, substantial heterogeneity in cultivar, growing substrate, harvest stage, extraction, and analytical methodology across the primary literature constrains direct quantitative comparison, so the concentration ranges compiled here should be read as indicative rather than definitive. These limitations temper the strength of the conclusions and reinforce the research priorities outlined above.

## 14. Conclusions

*Salicornia europaea* L. emerges from this review as a marine halophyte with a coherent bioactive profile that integrates several mechanistically complementary pathways relevant to human health. Its secondary metabolome—flavonols, isorhamnetin glycosides, hydroxycinnamic acids, oleanane-type triterpene saponins, fermentable polysaccharides, and a mineral-rich ionic matrix—supports antioxidant, anti-inflammatory, vascular-protective, anti-adipogenic, glycaemic-modulating, and microbiome-related effects through pathways involving NF-κB, PPAR-γ, endothelial nitric oxide signalling, and short-chain fatty acid production. Within this multi-target framework, the partial substitution of dietary sodium represents one applied dimension among several, rather than the central rationale for the ingredient.

The current evidence base remains predominantly mechanistic and preclinical, with consistent in vitro and animal data but a limited number of adequately powered human intervention trials. Pharmacokinetic characterisation of key constituents in food matrices, standardisation of bioactive content across cultivars and post-harvest processing, and metagenomics-level evaluation of the microbiome response are among the most actionable research priorities. Within these constraints, *S. europaea* can be reasonably described as a promising marine-derived functional ingredient whose translational value rests on the convergence of a multi-mechanistic bioactive profile, an established history of food use across several jurisdictions, and a sustainable agronomic footprint. Continued progress will depend on the generation of clinical evidence aligned with the mechanistic breadth already documented in vitro and in animal models, ensuring that future applications in precision nutrition and functional food development are supported by robust human data.

## Figures and Tables

**Table 1:** Bioactive phytochemical composition of *Salicornia europaea* and related *Salicornia* spp. Values represent representative ranges from published literature (aerial parts, dry weight basis unless stated). dw = dry weight; GAE = gallic acid equivalents; TPC = total phenolic content; PPAR-γ = peroxisome proliferator-activated receptor gamma; SCFA = short-chain fatty acids.

Compound Class	Key Compounds	Concentration (dw)	Documented Bioactivities	Key References
*Flavonols*	Quercetin, kaempferol, luteolin, rutin, myricetin	3–8 mg/g	Antioxidant (DPPH/FRAP); NF-κB inhibition; LDL oxidation protection; endothelial function	[9,17,18]
*Isorhamnetin glycosides*	Isorhamnetin-3-*O*-glucoside; isorhamnetin-3-*O*-rutinoside	0.5–2 mg/g	Anti-adipogenic (PPAR-γ inhibition); lipid metabolism; anti-steatotic	[15,21]
*Phenolic acids*	Caffeic acid, ferulic acid, *p*-coumaric acid, protocatechuic acid, chlorogenic acid	8–15 mg GAE/g	Antioxidant; anti-inflammatory (COX-2); vascular protection (NO pathway); eNOS activation	[16,22,23]
*Oleanane triterpene saponins*	Oleanolic acid glycosides; hederagenin derivatives	Variable by species	Pancreatic lipase inhibition; bile acid binding; anti-adipogenic; anti-inflammatory	[24,25]
*Chromones & other phenolics*	Chromone derivatives; apigenin; naringenin	Minor fractions	Antioxidant; enzyme inhibition; cytokine modulation	[18]
*Carotenoids*	*β*-carotene, zeaxanthin, lutein	0.1–0.5 mg/g	Antioxidant (lipid phase); photoprotective; provitamin A activity	[9,18]
Minerals (ionic form)	K, Mg, Ca, Fe, Zn (naturally balanced electrolyte matrix)	K: 800–1200; Mg: 200–400; Ca: 150–300; Fe: 40–80 mg/100 g dw	Electrolyte homeostasis; cardiovascular protection; 40–50% less bioavailable Na than NaCl; enzymatic cofactors	[8,9,16]
Dietary fibre	Cellulose, hemicellulose, pectin-like galacturonans, soluble mannans, arabinoxylans	15–25 g/100 g dw	Prebiotic (*Bifidobacterium*, *Lactobacillus*); SCFA production; cholesterol binding; glycaemic attenuation	[26,27]

Sources: Rodrigues et al. [17]; Patel [18]; Barreira et al. [9]; Kim et al. [16]; Park et al. [15]; Lv et al. [21]; Flint et al. [26]; Conlon & Bird [27].

**Table 2:** Summary of preclinical evidence for the cardiometabolic and health-promoting effects of *Salicornia europaea* and related species. SBP = systolic blood pressure; TPC = total phenolic content; PPAR-γ = peroxisome proliferator-activated receptor gamma; NF-κB = nuclear factor kappa-light-chain-enhancer of activated B cells; LPS = lipopolysaccharide; IC_50_ = half-maximal inhibitory concentration.

Study Type	Model	Extract/Form	Key Outcome	Effect Size/Finding	Ref
In vitro/ex vivo	*Isolated aortic rings; HUVECs*	*S. europaea aqueous extract*	No vascular dysfunction at equivalent Na load to NaCl; eNOS activation	Significant protection vs. NaCl control; ferulic acid identified as principal mediator	[16]
In vivo (animal)	Hypertensive rat model	*S. europaea extract (oral)*	Attenuation of high-salt-induced vascular dysfunction	SBP reduction ~15 mmHg vs. high-salt control	[16]
In vitro	3T3-L1 preadipocytes	Isorhamnetin-3-*O*-glucoside (isolated)	PPAR-γ and C/EBPα downregulation; lipid droplet inhibition	40–60% reduction in lipid accumulation at 10–50 μM; dose-dependent	[15]
In vitro	Pancreatic lipase enzyme assay	*S. europaea methanolic extract*	Lipase inhibitory activity (anti-obesity)	IC_50_ ~0.3 mg/mL	[25]
In vitro	RAW 264.7 macrophages (LPS-stimulated)	*S. herbacea polyphenolic extract*	NF-κB inhibition; TNF-α, IL-6, IL-1β reduction	Significant cytokine reduction vs. LPS control (*p* < 0.05)	[17,18]
In vitro (DPPH, FRAP, ORAC)	*Multiple Salicornia spp. extracts*	Aerial parts, various solvents	Strong free radical scavenging; TPC correlates with activity	DPPH IC_50_: 0.3–0.9 mg/mL; FRAP: 50–120 μmol Fe^2+^/g dw	[9,17,18]
In vitro	*α*-glucosidase enzyme assay	*Salicornia spp. flavonoid fractions*	*α*-glucosidase inhibitory activity (glycaemic control)	Quercetin IC_50_: 0.15–0.4 mg/mL; isorhamnetin active	[29,42]
In vivo (animal)	High-fat diet mouse model	*Isorhamnetin supplementation*	Attenuation of hepatic steatosis; lipogenesis inhibition	Reduced liver lipid accumulation; improved ALT/AST	[21]

Sources: Kim et al. [16]; Park et al. [15]; Rodrigues et al. [17]; Patel [18]; Lv et al. [21]; Boots et al. [29]; Hayden & Ghosh [30].

**Table 3:** Applications of *Salicornia europaea* across functional food and beverage matrices: technological functions, technical considerations, target health positioning, and representative product contexts.

Application Matrix	Function of *Salicornia*	Technical Considerations	Target Health Positioning	Representative Product Context
Functional condiment/salt substitute	1:1 NaCl replacement; marine mineral delivery; umami note	Free-flowing format; standardised NaCl equivalent; shelf stability	Sodium reduction; potassium & Mg contribution; cardiovascular-relevant reformulation	Seasoning blends, HoReCa salt, artisan condiments, culinary salts
Plant-based meat alternative	Sodium reduction; Mg/Zn fortification; prebiotic fibre	Processing stability; flavour compatibility; texture neutrality	Reduced Na; Mg contribution (energy metabolism); Zn (immunomodulation)	Plant-based burgers, sausages, nuggets; sport nutrition formats
Functional fruit preparation/preservation	Clean-label mineral electrolyte; potassium/sodium balance	Compatibility with acidic pH; pasteurisation stability; colour preservation	Potassium contribution; reduced Na vs. conventional; digestive health	Fruit-based probiotic preparations, compotes, chutney formats
Electrolyte beverage (functional water)	Natural electrolyte (Na, K, Mg) source; antioxidant microdose	Solubility in clear beverages; sensory threshold at functional dose	Electrolyte delivery; hydration-oriented formulation; post-exercise use context	Isotonic sports drinks, functional mineral water, wellness shots
Nutraceutical/dietary supplement	Standardised bioactive extract; polyphenol concentrate; mineral capsule	Bioactive standardisation (TPC, isorhamnetin); GMP compliance	Metabolic-relevant bioactives; antioxidant capacity; glycaemic-modulation hypothesis; authorised nutrient-specific claims	Capsules, sachets, functional tablets; anti-obesity formulations (Korea)

Sources: Barreira et al. [9]; Kim et al. [16]; He & MacGregor [38]; Ventura & Sagi [8]; WHO SHAKE [51].

**Table 4:** International regulatory status of *Salicornia europaea* and related *Salicornia* spp. as a food ingredient, salt substitute, and functional food component across major global markets. GCC = Gulf Cooperation Council; GRAS = Generally Recognised As Safe; NRV = nutrient reference value; EFSA = European Food Safety Authority.

Region	Regulatory Status	Legal Basis/Key Regulation	Notes on Current Use and Market
European Union	Traditional food (not Novel Food)	Reg. (EU) 2015/2283; Commission Reg. (EU) 432/2012 (health claims); long consumption history in UK, France, Netherlands	Directly usable in food products; no EFSA Novel Food dossier required; EFSA-authorised claims for K (blood pressure), Mg (energy, muscle function), Ca (bones) achievable at functional doses
Japan	Traditional food (akkeshiso)	Food Sanitation Act; long coastal consumption history; FOSHU (Foods for Specified Health Uses) pathway available	Anti-fatigue capsules; mineral salt alternatives; functional food ingredient; documented use in coastal regions
South Korea	Traditional food (hamcho)	Korean Food Standards Codex; centuries of therapeutic and culinary use; National Institute of Food and Drug Safety Evaluation approval	Nutraceutical powders, glycaemic control supplements, isotonic and hangover recovery beverages; most developed clinical research base globally
China	Emerging food ingredient	GB 2760 Food Additive Standard; Ministry of Agriculture saline cultivation programmes; ongoing regulatory harmonisation	Bio-salt products; polyphenol extracts; anti-obesity supplement development; large-scale saline land cultivation initiatives in Shandong, Xinjiang
GCC (UAE, KSA, Bahrain)	Food ingredient (no specific restriction)	GSO 2336; equivalence to European samphire recognised; halal-compatible (plant origin); ICBA cultivation validation	Functional condiments; cardiovascular-relevant products; desert agroecology crop
USA/Canada	GRAS-equivalent (specialty vegetable)	FDA GRAS self-determination pathway; available as specialty vegetable/seasoning; Health Canada General Chapter provisions	Salt-reduction applications; functional food ingredient; FDA GRAS notification pathway available for extracts

Sources: Regulation (EU) 2015/2283 [53]; Commission Regulation (EU) 432/2012 [52]; WHO SHAKE [51].

## Data Availability

No new data were created or analyzed in this study. Data sharing is not applicable to this article.

## Abbreviations

The following abbreviations are used in this manuscript:

NCDs	non-communicable diseases
CVD	cardiovascular disease
T2DM	type 2 diabetes mellitus
WHO	World Health Organization
ROS	reactive oxygen species
UV	ultraviolet
TPC	total phenolic content
TFC	total flavonoid content
GAE	gallic acid equivalents
DPPH	2,2-diphenyl-1-picrylhydrazyl
FRAP	ferric reducing antioxidant power
LDL	low-density lipoprotein
HDL	high-density lipoprotein
SCFA	short-chain fatty acids
PPAR-γ	peroxisome proliferator-activated receptor gamma
NF-κB	nuclear factor kappa-light-chain enhancer of activated B cells
eNOS	endothelial nitric oxide synthase
NO	nitric oxide
ACE	angiotensin-converting enzyme
NAFLD	non-alcoholic fatty liver disease
HPLC-DAD	high-performance liquid chromatography with diode-array detection
LC-MS/MS	liquid chromatography–tandem mass spectrometry
NMR	nuclear magnetic resonance
HER	human epidermal growth factor receptor
AOM/DSS	azoxymethane/dextran sulphate sodium
RCT	randomized controlled trial
EFSA	European Food Safety Authority
NRV	nutrient reference value
